# Supplementary material for: Influence of the Method of Fe Deposition on the Surface of Hydrolytic Lignin on the Activity in the Process of Its Conversion in the Presence of CO2
Source: Int J Mol Sci. 2023 Jan 9;24(2):1279. doi: 10.3390/ijms24021279 (PMC9866296; doi:10.3390/ijms24021279)
Supplement: Supplementary file 1 [file ijms-24-01279-s001.zip › ijms-2060068-supplementary.pdf]

sSupporting information (SI) for the article

Influence of the method of Fe deposition on the surface of hydrolytic lignin on the activity in the process of its conversion in the presence of CO_2

Artem A. Medvedev [1,*], Alexander L. Kustov [1,2,3,*], Daria A. Beldova [1], Konstantin B. Kalmykov [1], Mikhail Yu. Mashkin [1], Anastasia A. Shesterkina [1,2,3], Sergey F. Dunaev [1] and Leonid M. Kustov [1,2,3]

[1] Chemistry Department, Moscow State University, 119992, Moscow Russia

[2] National University of Science and Technology MISiS, 119071, Moscow, Russia

[3] N. D. Zelinsky Institute of Organic Chemistry, Russian Academy of Sciences, 119991, Moscow, Russia

Corresponding authors: artom.medvedev@yandex.ru (Medvedev A. A.), kyst@list.ru (Kustov A. L.)

Table S1. Elemental analysis of the starting hydrolytic lignin by CHNOS analyzer.

Element	C, wt. %	O, wt. %	H, wt. %	N, wt. %	S, wt. %	Ash, wt. %
Content	52.82	31.91	5.34	0.36	0.18	9.41

As can be seen from the table above, the used lignin demonstrates a relatively high ash content, which can be the reason for the “internal” (own) catalytic activity of lignin in the investigated reaction because of the metal content. The results of the catalytic tests with not impregnated lignin demonstrate such an activity, but it is not so high.

Table S2. The results of measuring the moisture capacity of hydrolytic lignin with various solvents.

Solvent	H_2O	*i*PrOH	EtOH	Acetone
Moisture capacity, ml/g	3.93	5.52	5.25	6.10

EDX results

<u>LN-1H_2O</u> (*Impregnated with an aqueous solution of iron (III) nitrate by incipient wetness*)

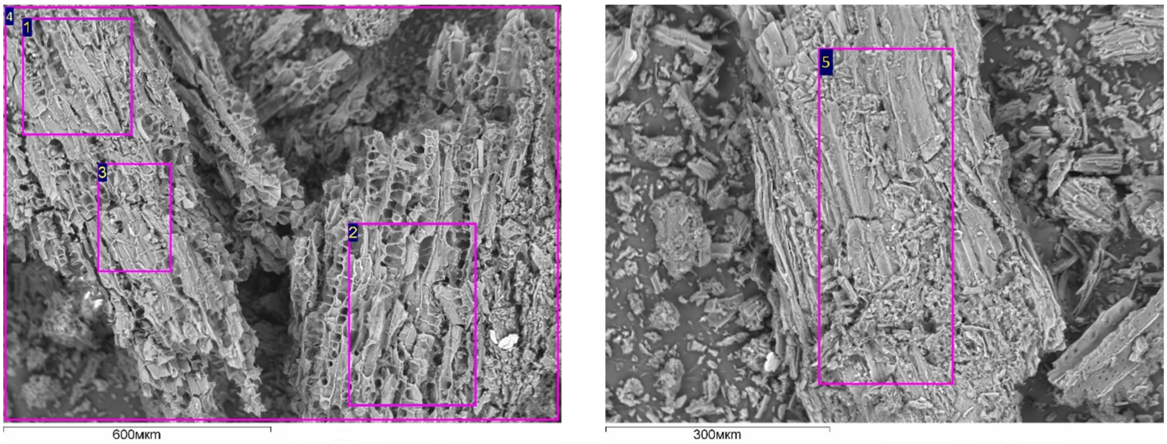

Figure S1. Microphotos of the sample LN-1H_2O. EDX data were collected from the marked areas.

Table S3. EDX data for the sample LN-1H_2O.

Entry	C, wt. %	O, wt. %	Al, wt. %	Si, wt. %	S, wt. %	Ca, wt. %	Fe, wt. %
1	57.1	35.0	0.01	0.0	0.4	0.5	7.1
2	58.3	36.3	0.04	0.0	0.3	0.4	4.7
3	58.3	33.0	0.02	0.0	0.4	0.4	7.8
4	55.5	34.3	0.05	0.1	0.3	0.6	9.2
5	56.3	32.2	0.03	0.2	0.5	0.9	10.0
Average	57.1	34.1	0.03	0.05	0.4	0.6	7.8
SD	1.22	1.62	0.02	0.07	0.06	0.19	2.05

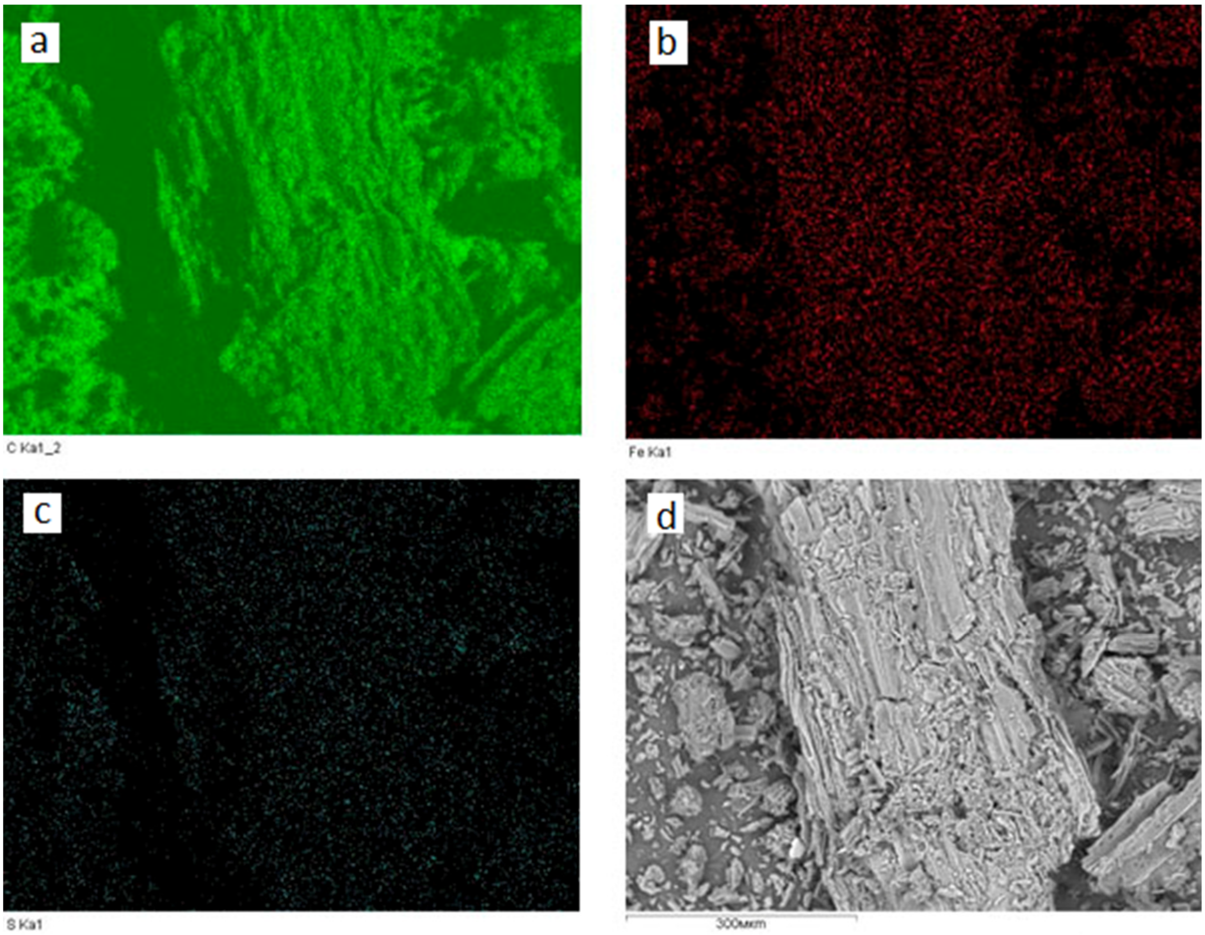

Figure S2. Element maps by EDX for the sample LN-1H_2O.

LN-2H_2O (*Impregnated with an aqueous solution of iron (III) nitrate by twofold incipient wetness*)

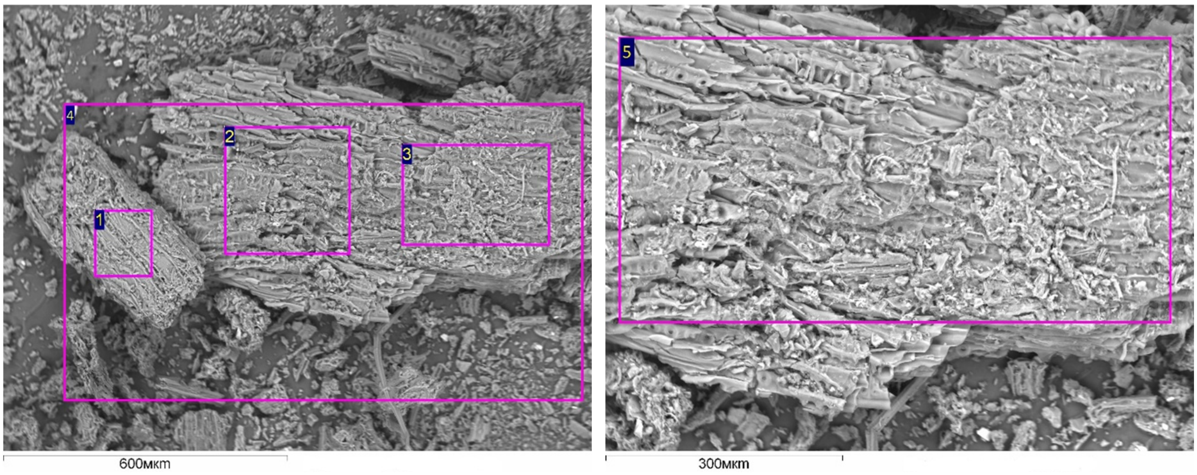

Figure S3. Microphotos of the sample LN-2H_2O. EDX data were collected from the marked areas.

Table S4. EDX data for the sample LN-2H_2O.

Entry	C, wt. %	O, wt. %	Al, wt. %	Si, wt. %	S, wt. %	Ca, wt. %	Fe, wt. %
1	55.4	35.7	0.1	0.2	0.4	0.6	7.8
2	61.8	31.2	0.1	0.3	0.3	0.5	5.8
3	59.7	33.9	0.1	0.2	0.3	0.5	5.3
4	55.2	37.6	0.1	0.1	0.4	0.4	6.2
5	63.4	32.3	0.0	0.1	0.2	0.3	3.7
Average	59.1	34.1	0.1	0.2	0.3	0.5	5.8
SD	3.74	2.56	0.03	0.10	0.07	0.10	1.48

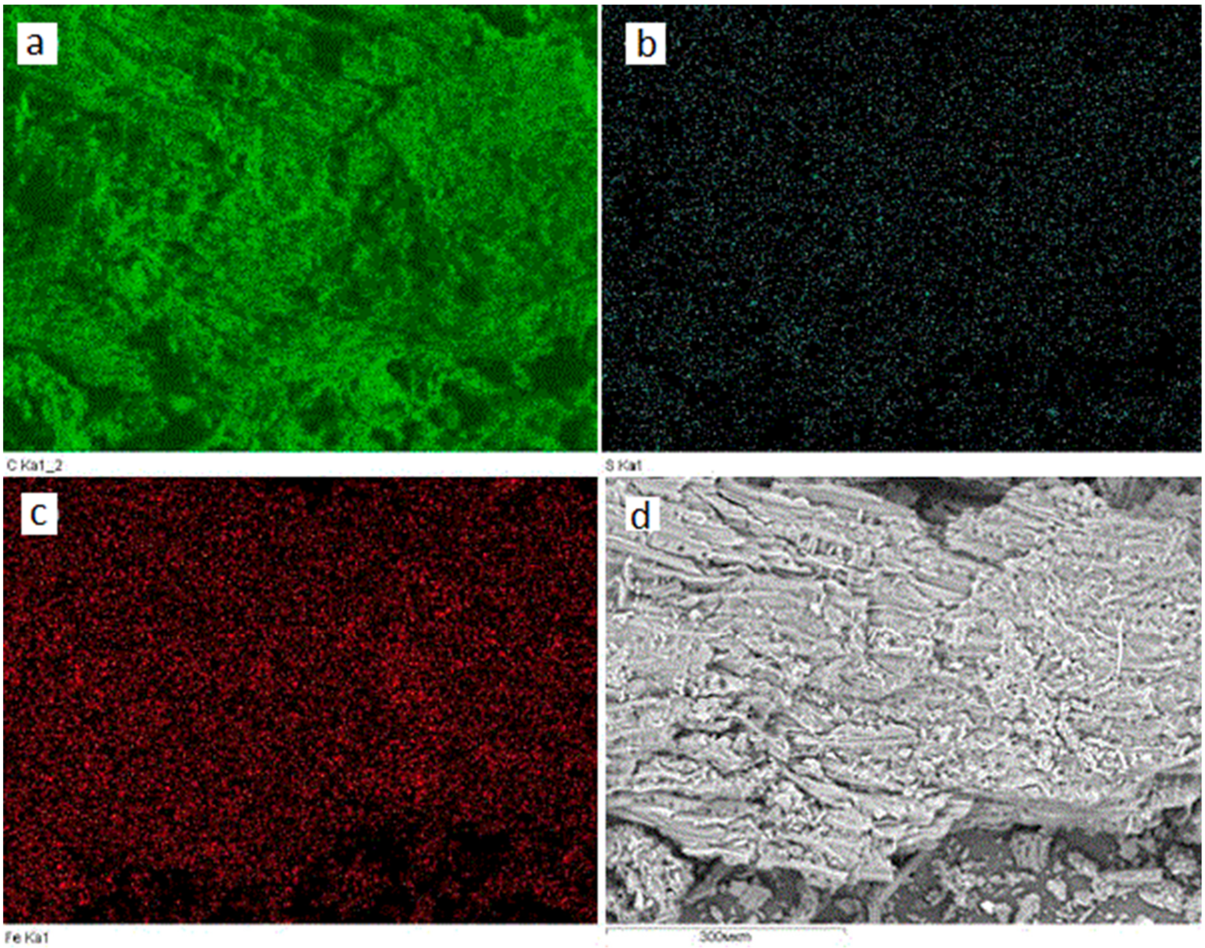

Figure S4. Element maps by EDX for the sample LN-2H_2O.

LN-i*PrOH* (*Impregnated with a solution of iron (III) nitrate in isopropanol by incipient wetness*)

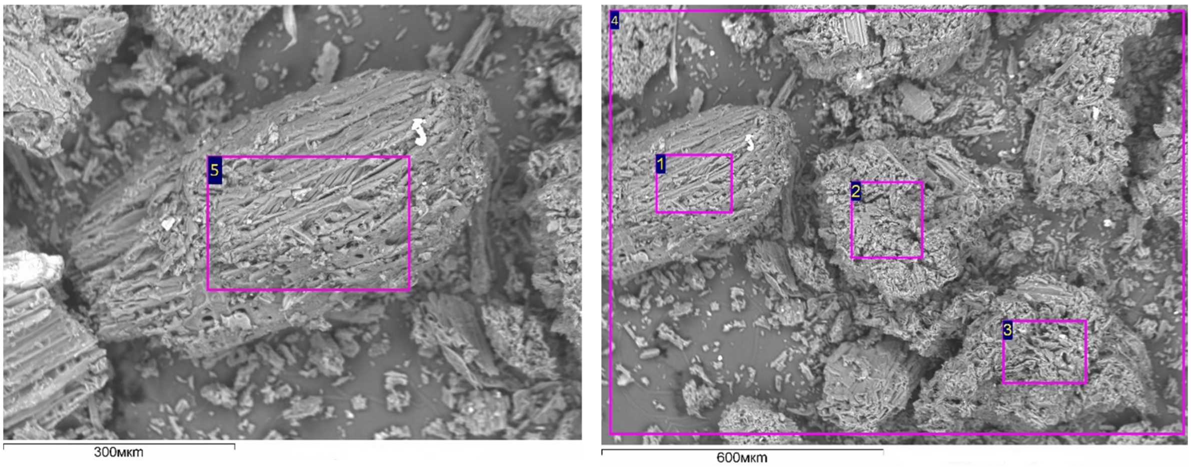

Figure S5. Microphotos of the sample LN-*i*PrOH. EDX data were collected from the marked areas.

Table S5. EDX data for the sample LN-*i*PrOH.

Entry	C, wt. %	O, wt. %	Al, wt. %	Si, wt. %	S, wt. %	Ca, wt. %	Fe, wt. %
1	58.6	33.1	0.2	0.1	0.5	0.4	7.0
2	59.8	35.1	0.1	0.4	0.3	0.3	4.1
3	56.7	37.9	0.1	0.9	0.2	0.3	3.9
4	55.2	35.5	0.3	0.2	0.3	0.4	8.1
5	60.3	33.9	0.5	0.2	0.4	0.4	4.3
Average	58.1	35.1	0.2	0.4	0.3	0.4	5.5
SD	2.14	1.82	0.15	0.34	0.12	0.07	1.95

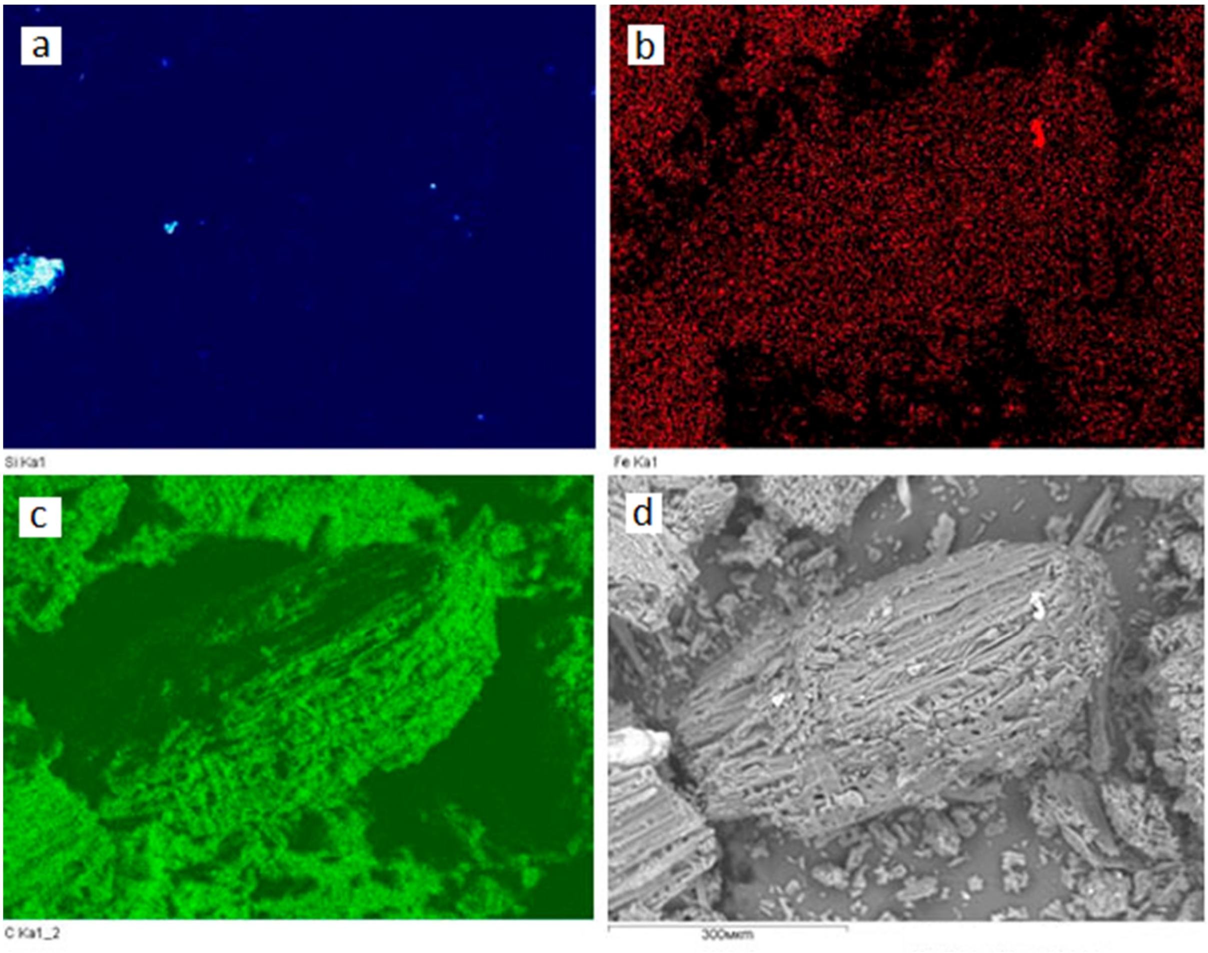

Figure S6. Element maps by EDX for the sample LN-*i*PrOH.

<u>LN-acetone</u> (*Impregnated with a solution of iron (III) nitrate in acetone by incipient wetness*)

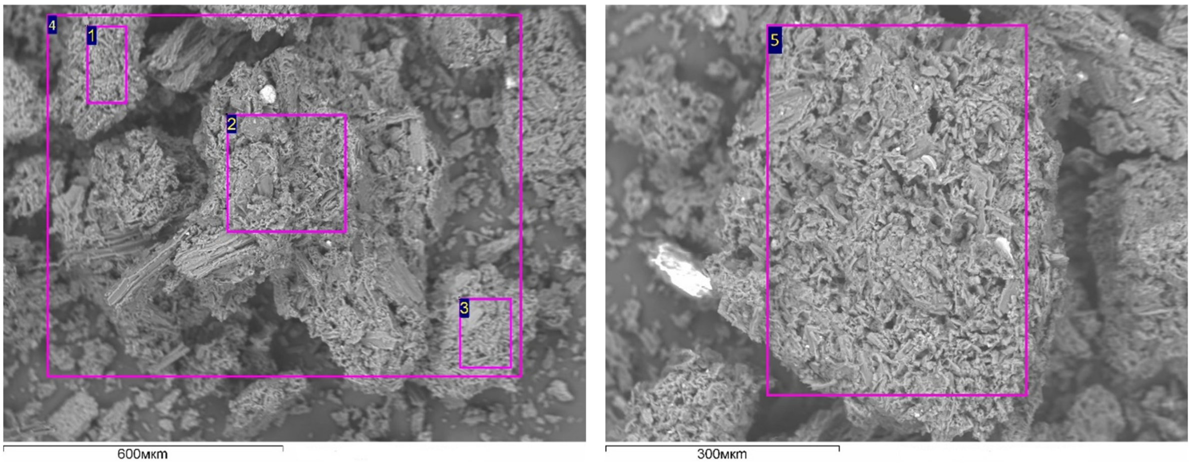

Figure S7. Microphotos of the sample LN-acetone. EDX data were collected from the marked areas.

Table S6. EDX data for the sample LN-acetone.

Entry	C, wt. %	O, wt. %	Al, wt. %	Si, wt. %	S, wt. %	Ca, wt. %	Fe, wt. %
1	57.8	36.4	0.1	0.3	0.3	0.3	4.8
2	55.0	39.2	0.1	0.3	0.3	0.3	4.9
3	55.2	35.9	0.1	0.1	0.4	0.5	7.8
4	56.7	35.6	0.0	0.1	0.3	0.6	6.8
5	57.2	35.3	0.1	0.2	0.3	0.4	6.5
Average	56.4	36.5	0.1	0.2	0.3	0.4	6.1
SD	1.24	1.55	0.03	0.10	0.06	0.11	1.31

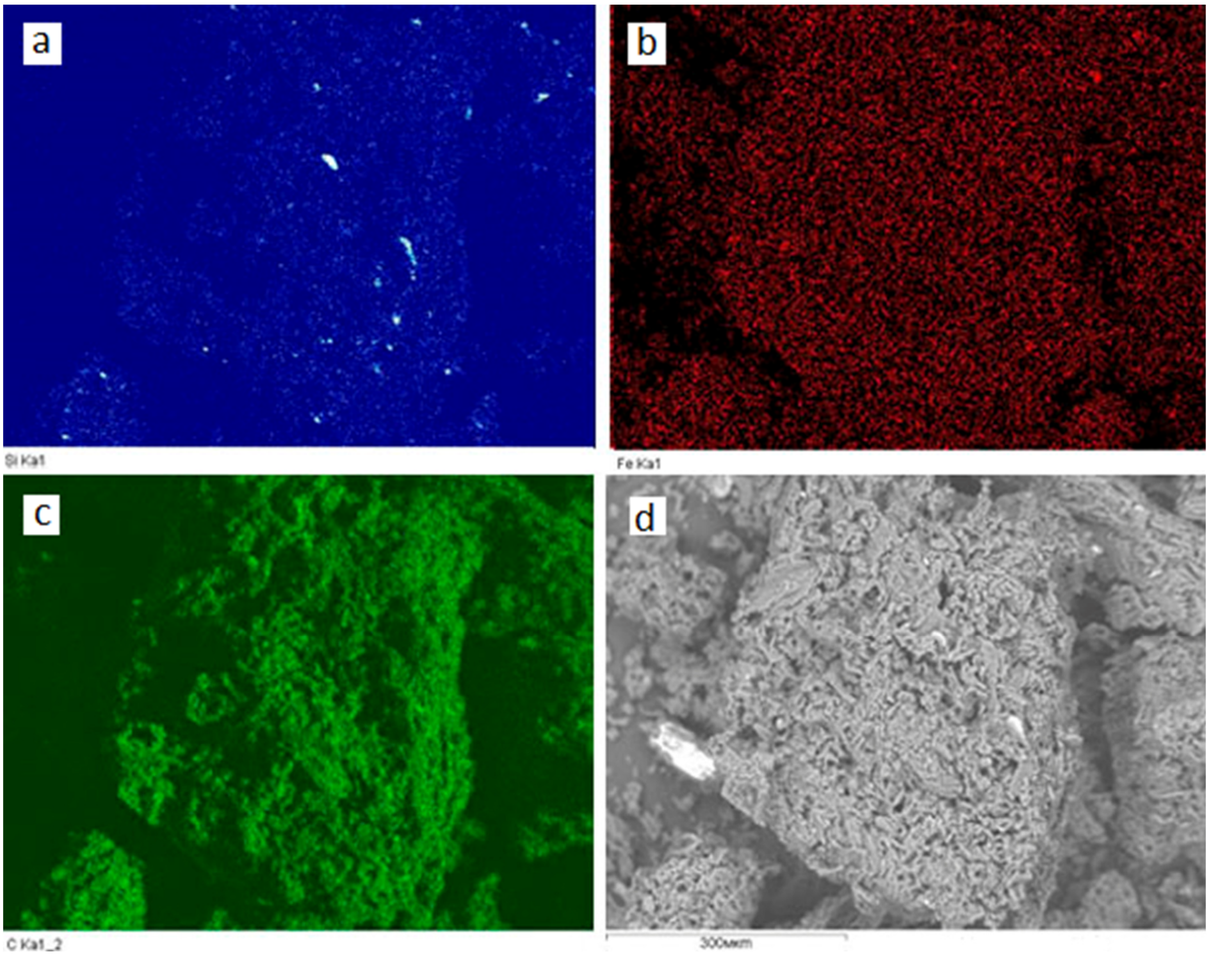

Figure S8. Element maps by EDX for the sample LN-acetone.

LN-ethanol (*Impregnated with a solution of iron (III) nitrate in ethanol by incipient wetness*)

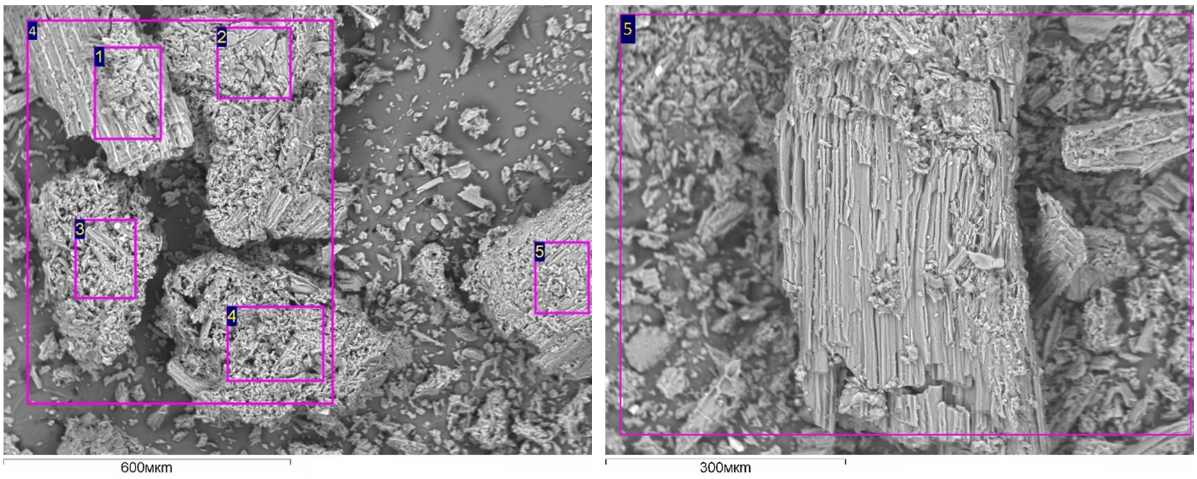

Figure S9. Microphotos of the sample LN-ethanol. EDX data were collected from the marked areas.

Table S7. EDX data for the sample LN-ethanol.

Entry	C, wt. %	O, wt. %	Al, wt. %	Si, wt. %	S, wt. %	Ca, wt. %	Fe, wt. %
1	56.4	31.2	0.1	0.1	0.3	0.6	11.4
2	55.1	38.4	0.1	0.1	0.2	0.4	5.8
3	56.1	37.0	0.2	0.2	0.4	0.5	5.8
4	56.4	37.7	0.1	0.2	0.3	0.4	5.0
5	56.9	37.5	0.0	0.1	0.2	0.4	4.9
Average	56.2	36.4	0.1	0.2	0.3	0.4	6.6
SD	0.69	2.91	0.05	0.04	0.10	0.11	2.72

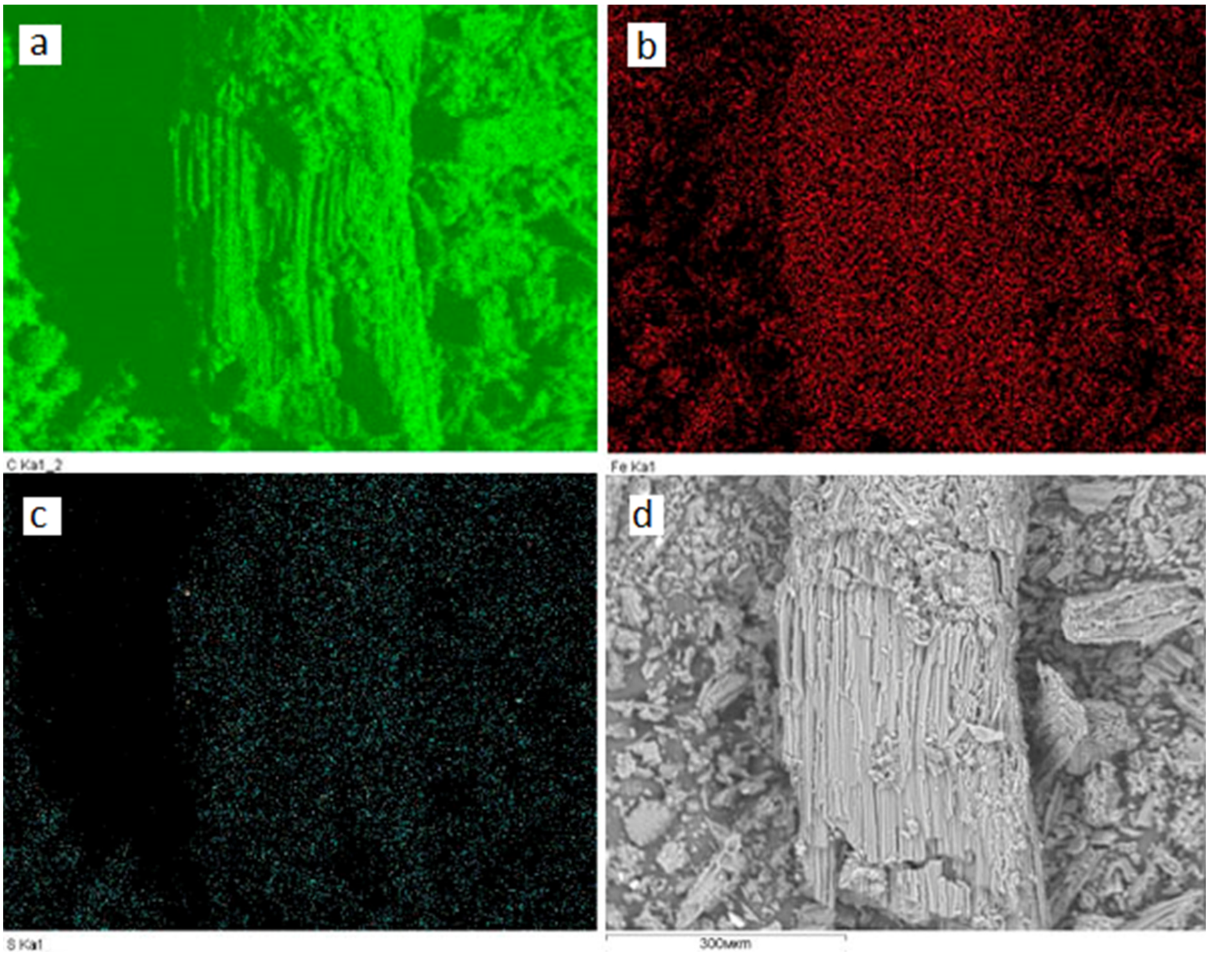

Figure S10. Element maps by EDX for the sample LN-ethanol.

LS-III (*Impregnated with a solution of iron (III) sulfate in water by incipient wetness*)

The formation of crystals of iron (III) sulfate on the surface of lignin can be seen in the SEM image.

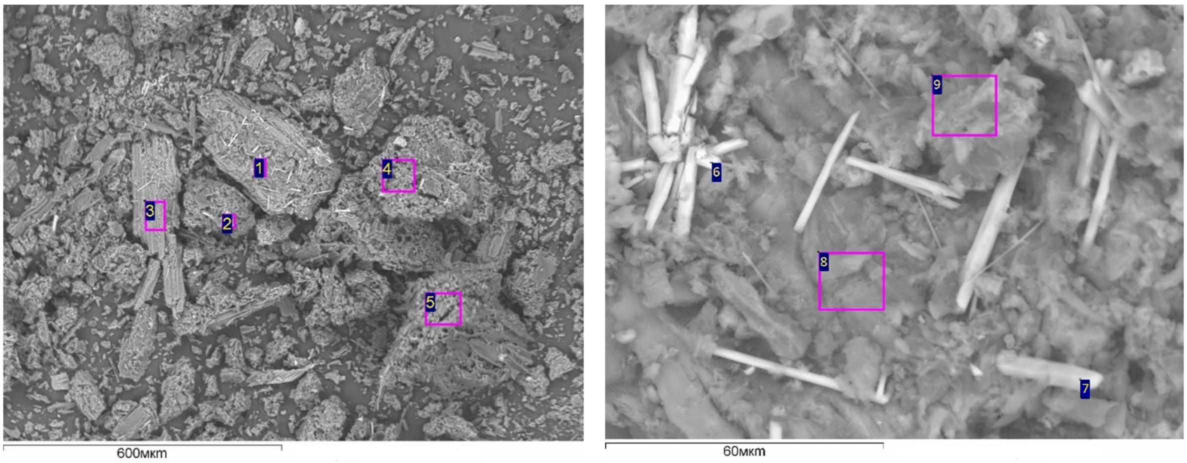

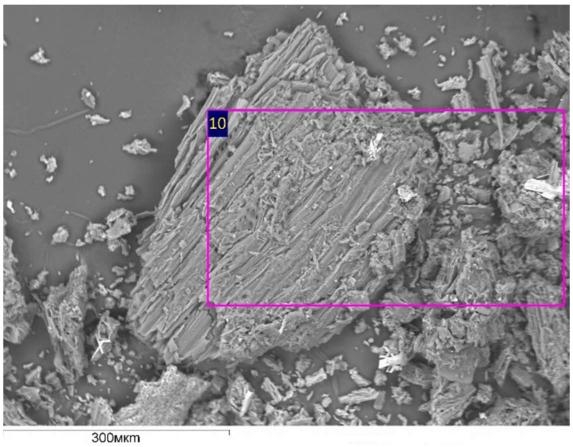

Figure S11. Microphotos of the sample LS-III. EDX data were collected from the marked areas.

Table S8. EDX data for the sample LS-III.

Entry	C, wt. %	O, wt. %	Al, wt. %	Si, wt. %	S, wt. %	Ca, wt. %	Fe, wt. %
1	49.3	29.1	0.1	0.1	9.5	7.9	4.1
2	28.7	43.7	0.0	0.0	12.7	13.4	1.5
3	33.4	11.5	0.1	0.1	9.5	0.1	45.4
4	50.9	32.4	0.0	0.2	6.9	0.5	9.0
5	51.4	35.4	0.1	0.1	6.0	0.1	6.8
7	48.2	29.1	0.0	0.0	8.9	11.1	2.7
8	45.1	28.0	0.1	0.0	11.4	14.4	1.1
9	51.0	31.5	0.1	0.3	7.1	0.2	9.9
10	64.2	20.2	0.1	0.0	6.7	0.1	8.7
Average	46.9	29.0	0.1	0.1	8.8	5.3	9.9
SD	10.47	9.10	0.03	0.09	2.27	6.29	13.72

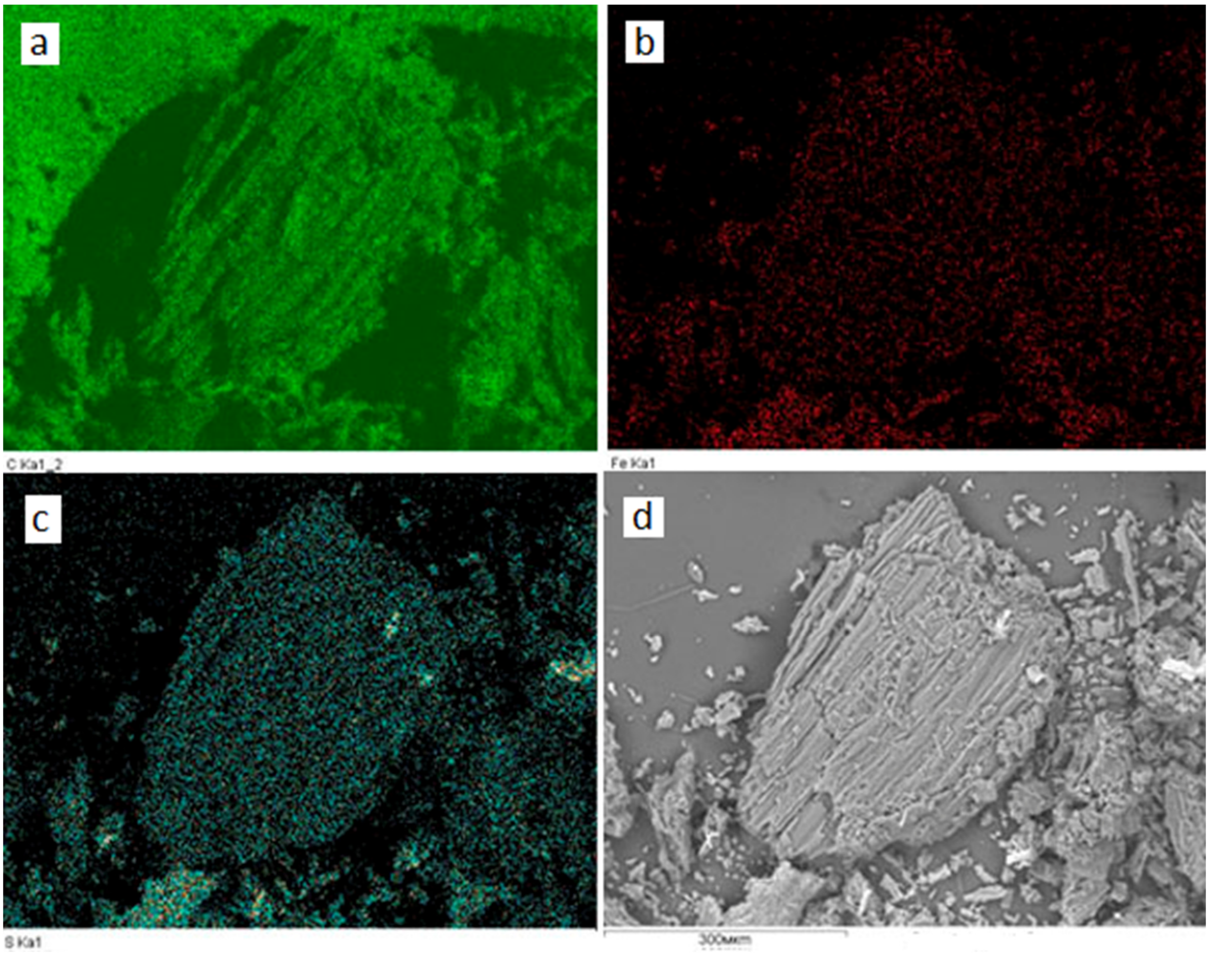

Figure S12. Element maps by EDX for the sample LS-III.

<u>LS-II</u> (*Impregnated with a solution of iron (II) sulfate in water by incipient wetness*)

A coating over the surface of lignin can be seen in SEM images. The white areas coincide with areas with high iron content.

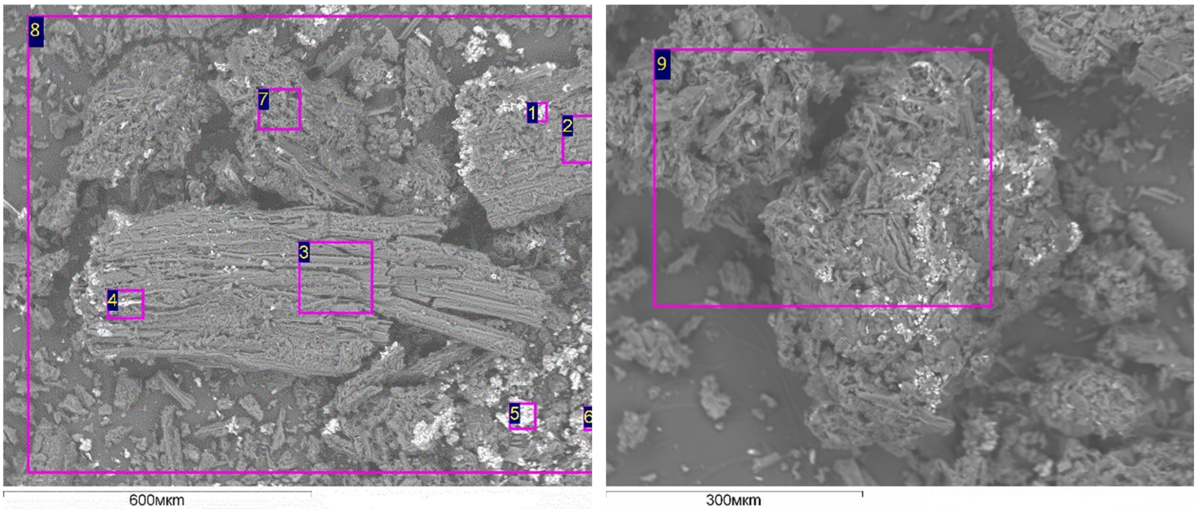

Figure S13. Microphotos of the sample LS-II. EDX data were collected from the marked areas.

Table S9. EDX data for the sample LS-II.

Entry	C, wt. %	O, wt. %	Al, wt. %	Si, wt. %	S, wt. %	Ca, wt. %	Fe, wt. %
1	35.8	40.3	0.0	0.1	7.8	0.0	15.9
2	61.5	33.4	0.1	0.4	1.6	0.1	2.9
3	56.4	38.9	0.0	0.1	1.8	0.0	2.8
4	54.4	34.6	0.0	0.1	3.7	0.1	7.1
5	24.3	30.9	0.0	0.0	13.4	0.0	31.4
7	32.8	41.3	0.0	0.1	9.2	0.1	16.6
8	61.1	33.8	0.2	0.5	1.5	0.1	2.8
9	63.6	30.0	0.1	0.4	1.7	0.2	4.1
Average	49.9	35.4	0.1	0.2	4.7	0.1	9.7
SD	14.80	4.02	0.07	0.17	4.36	0.05	9.85

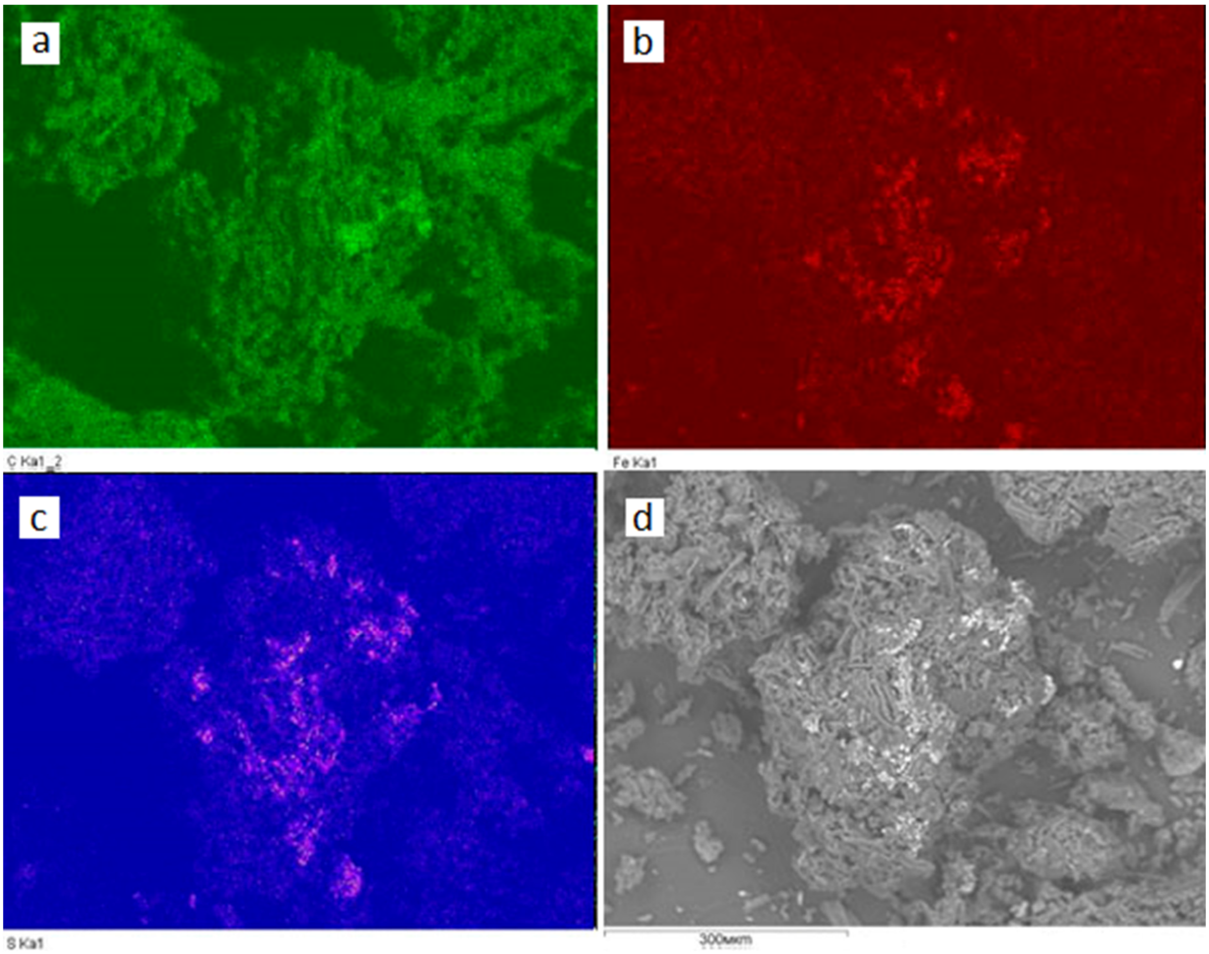

Figure S14. Element maps by EDX for the sample LS-II.
